# 10-Methyl-9-[2-(propan-2-yl)phenoxy­carbonyl]­acridinium trifluoro­methane­sulfonate

**DOI:** 10.1107/S160053681003953X

**Published:** 2010-10-09

**Authors:** Damian Trzybiński, Karol Krzymiński, Jerzy Błażejowski

**Affiliations:** a Faculty of Chemistry, University of Gdańsk, J. Sobieskiego 18, 80-952 Gdańsk, Poland

## Abstract

In the crystal of the title compound, C_24_H_22_NO_2_^+^·CF_3_SO_3_^−^, adjacent cations and anions are connected through C—H⋯O, C—H⋯F and S–O⋯π inter­actions, while neighboring cations *via* π–π inter­actions [centroid–centroid distance = 3.962 (2) Å]. The acridine and benzene ring systems are oriented at a dihedral angle of 14.6 (1)°. The carboxyl group is twisted at an angle of 87.6 (1)° relative to the acridine skeleton. The mean planes of adjacent acridine units are parallel or inclined at an angle of 13.4 (1)° in the crystal structure.

## Related literature

For background to the chemiluminogenic properties of 9-phen­oxy­carbonyl-10-methyl­acridinium trifluoro­meth­ane­sulfonates, see: Natrajan *et al.* (2010 ▶); Brown *et al.* (2009 ▶); King *et al.* (2007 ▶); Rak *et al.* (1999 ▶); Roda *et al.* (2003 ▶); Zomer & Jacquemijns (2001 ▶). For related structures, see: Sikorski *et al.* (2006 ▶, 2007 ▶); Trzybiński *et al.* (2010 ▶). For inter­molecular inter­actions, see: Bianchi *et al.* (2004 ▶); Dorn *et al.* (2005 ▶); Hunter *et al.* (2001 ▶); Lyssenko & Anti­pin (2004 ▶); Novoa *et al.* (2006 ▶). For the synthesis, see: Sato (1996 ▶); Trzybiński *et al.* (2010 ▶).
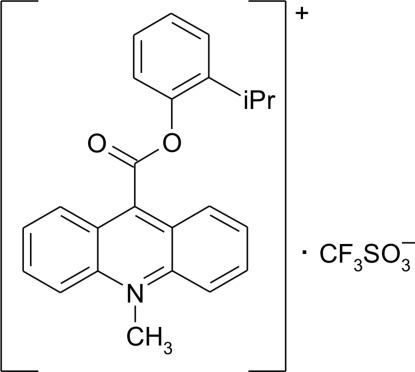


## Experimental

### 

#### Crystal data

C_24_H_22_NO_2_^+^·CF_3_SO_3_^−^
*M* *_r_* = 505.51
Monoclinic, 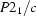

*a* = 14.4346 (7) Å
*b* = 12.9677 (5) Å
*c* = 13.0862 (5) Å
β = 107.160 (5)°
*V* = 2340.47 (17) Å^3^
*Z* = 4
Mo *K*α radiation
μ = 0.20 mm^−1^
*T* = 295 K
0.32 × 0.20 × 0.05 mm

#### Data collection

Oxford Diffraction Gemini R Ultra Ruby CCD diffractometer
Absorption correction: multi-scan (*CrysAlis RED*; Oxford Diffraction, 2008 ▶) *T*_min_ = 0.955, *T*_max_ = 1.000
17556 measured reflections
4169 independent reflections
2436 reflections with *I* > 2σ(*I*)
*R*_int_ = 0.043

#### Refinement

*R*[*F* ^2^ > 2σ(*F* ^2^)] = 0.045
*wR*(*F* ^2^) = 0.116
*S* = 0.93
4169 reflections
319 parameters
H-atom parameters constrained
Δρ_max_ = 0.34 e Å^−3^
Δρ_min_ = −0.26 e Å^−3^

### 

Data collection: *CrysAlis CCD* (Oxford Diffraction, 2008 ▶); cell refinement: *CrysAlis RED* (Oxford Diffraction, 2008 ▶); data reduction: *CrysAlis RED*; program(s) used to solve structure: *SHELXS97* (Sheldrick, 2008 ▶); program(s) used to refine structure: *SHELXL97* (Sheldrick, 2008 ▶); molecular graphics: *ORTEP-3* (Farrugia, 1997 ▶); software used to prepare material for publication: *SHELXL97* and *PLATON* (Spek, 2009 ▶).

## Supplementary Material

Crystal structure: contains datablocks global, I. DOI: 10.1107/S160053681003953X/ng5040sup1.cif

Structure factors: contains datablocks I. DOI: 10.1107/S160053681003953X/ng5040Isup2.hkl

Additional supplementary materials:  crystallographic information; 3D view; checkCIF report

## Figures and Tables

**Table 1:** Hydrogen-bond geometry (Å, °)

*D*—H⋯*A*	*D*—H	H⋯*A*	*D*⋯*A*	*D*—H⋯*A*
C4—H4⋯O29^i^	0.93	2.48	3.363 (3)	159
C27—H27*C*⋯F35^i^	0.96	2.51	3.250 (4)	134

Symmetry code: (i) 
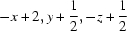
.

**Table 2:** S–O⋯π inter­actions (Å,°) *Cg*2 is the centroids of the C1–C4/C11/C12 ring.

*X*	*I*	*J*	*I*⋯*J*	*X*⋯*J*	*X*–*I*⋯*J*
S28	O31	*Cg*2^ii^	3.208 (2)	4.128 (2)	120.3 (2)

Symmetry code: (ii) 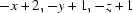.

**Fig. 1.**
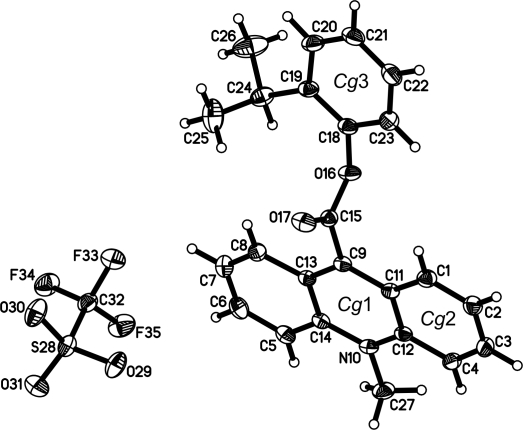
The molecular structure of the title compound showing the atom-labeling scheme. Displacement ellipsoids are drawn at the 25% probability level and H atoms are shown as small spheres of arbitrary radius. Cg1, Cg2, and Cg3 denote the ring centroids.

**Fig. 2.**
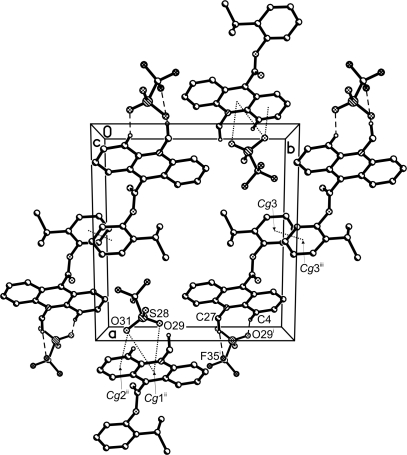
The arrangement of the ions in the crystal structure. The C–H···O and C–H···F interactions are represented by dashed lines, the S–O···π and π–π contacts by dotted lines. H atoms not involved in interactions have been omitted. [Symmetry codes: (i) –x + 2, y + 1/2, –z + 1/2; (ii) –x + 2, –y + 1, –z + 1; (iii) –x + 1, –y + 2, –z + 1.]

## supplementary crystallographic information

### Comment

9-(Phenoxycarbonyl)-10-methylacridinium salts have long been known as chemiluminescent indicators or the chemiluminogenic fragments of chemiluminescent labels widely used in assays of biologically and environmentally important entities such as antigens, antibodies, enzymes or DNA fragments (Zomer & Jacquemijns, 2001; Roda *et al.*, 2003; King *et al.*, 2007; Brown *et al.*, 2009; Natrajan *et al.*, 2010). The cations of these salts are oxidized with H_2_O_2_ in alkaline media, a reaction that produces light. The latter process is accompanied by the removal of the phenoxycarbonyl fragment and the conversion of the remaining part of the molecules to electronically excited, light-emitting 10-methyl-9-acridinone (Rak *et al.*, 1999). The efficiency of chemiluminescence – crucial for analytical applications – is affected by the structure of the phenyl fragment (Zomer & Jacquemijns, 2001; Natrajan *et al.*, 2010). In the search for efficient chemiluminogens we synthesized 9-(phenoxycarbonyl)-10-methylacridinium derivatives alkyl substituted in the *ortho* position of the phenyl fragment. Here we present the structure of 9-(2-*i*-propylphenoxycarbonyl)-10-methylacridinium trifluoromethanesulfonate.

In the cation of the title compound (Fig. 1), the bond lengths and angles characterizing the geometry of the acridinium moiety are typical of acridine-based derivatives (Sikorski *et al.*, 2006; Sikorski *et al.*, 2007; Trzybiński *et al.*, 2010). With respective average deviations from planarity of 0.0127 (3) Å and 0.0030 (3) Å, the acridine and benzene ring systems are oriented at a dihedral angle of 14.6 (1)°. The carboxyl group is twisted at an angle of 87.6 (1)° relative to the acridine skeleton. The mean planes of the adjacent acridine moieties are parallel (remain at an angle 0.0 (1)°) or inclined at an angle of 87.6 (1)° in the lattice. In the series of 9-phenoxycarbonyl-10-methylacridinium trifluoromethanesulfonates substituted in the *ortho* position of the phenyl fragment with Me (Sikorski *et al.*, 2006), Et (Trzybiński *et al.*, 2010), *i*-Pr (this work) and *t*-Bu (Sikorski *et al.*, 2007), the dihedral angle between acridine and the benzene ring, and that between the carboxyl group and the acridine skeleton, increase in the order 2-Et < 2-*i*-Pr < 2-Me < 2-*t*-Bu, and 2-*t*-Bu < 2-Et < 2-*i*-Pr < 2-Me, respectively. This implies that increasing size of the alkyl substituent in the *ortho* position does not systematically influence the mutual arrangement of the above mentioned fragments of the molecules.

In the crystal structure, each anion is connected to the adjacent cations through C–H···O (Table 1, Fig. 2), C–H···F (Table 1, Fig. 2) and S–O···π  (Table 2, Fig. 2) interactions. Neighboring cations contact each other via π–π (Table 3, Fig. 2) interactions. The C–H···O (Novoa *et al.* 2006) and C–H··· F (Bianchi *et al.*, 2004; Lyssenko & Antipin, 2004) interactions are of the hydrogen bond type. The S–O···π (Dorn *et al.*, 2005) and the π–π (Hunter *et al.*, 2001) interactions should be of an attractive nature. The crystal structure is stabilized by a network of these short-range specific interactions and by long-range electrostatic interactions between ions.

### Experimental

9-(Chlorocarbonyl)acridine, obtained by treating acridine-9-carboxylic acid with a tenfold molar excess of thionyl chloride, was first esterified with 2-*i*-propylphenol in anhydrous dichloromethane in the presence of *N*,*N*-diethylethanamine and a catalytic amount of *N*,*N*-dimethyl-4-pyridinamine (room temperature, 15h) (Sato, 1996) to obtain 2-*i*-propylphenylacridine-9-carboxylate (purified chromatographically (SiO_2_, cyclohexane/ethyl acetate, 1/1 v/v)). The latter compound was then quaternarized with a fivefold molar excess of methyl trifluoromethanesulfonate dissolved in anhydrous dichloromethane (Trzybiński *et al.*, 2010). The crude 9-(*i*-propylphenoxycarbonyl)-10-methylacridinium trifluoromethanesulfonate was dissolved in a small amount of ethanol, filtered and precipitated with 20 v/v excess of diethyl ether. Yellow crystals suitable for X-ray investigations were grown from absolute ethanol solution (m.p. 464–466 K).

### Refinement

H atoms were positioned geometrically, with C—H = 0.93 Å and 0.96 Å for the aromatic and alkyl H atoms, respectively, and constrained to ride on their parent atoms with *U*_iso_(H) = *xU*_eq_(C), where *x* = 1.2 for the aromatic and *x* = 1.5 for the alkyl H atoms.

### Crystal data

**Table:**

C_24_H_22_NO_2_^+^·CF_3_SO_3_^−^	*F*(000) = 1048
*M**_r_* = 505.51	*D*_x_ = 1.435 Mg m^−^^3^
Monoclinic, *P*2_1_/*c*	Mo *K*α radiation, λ = 0.71073 Å
Hall symbol: -P 2ybc	Cell parameters from 5919 reflections
*a* = 14.4346 (7) Å	θ = 3.0–29.2°
*b* = 12.9677 (5) Å	µ = 0.20 mm^−^^1^
*c* = 13.0862 (5) Å	*T* = 295 K
β = 107.160 (5)°	Prism, yellow
*V* = 2340.47 (17) Å^3^	0.32 × 0.20 × 0.05 mm
*Z* = 4	

### Data collection

**Table:**

Oxford Diffraction Gemini R Ultra Ruby CCD diffractometer	4169 independent reflections
Radiation source: Enhanced (Mo) X-ray Source	2436 reflections with *I* > 2σ(*I*)
graphite	*R*_int_ = 0.043
Detector resolution: 10.4002 pixels mm^-1^	θ_max_ = 25.1°, θ_min_ = 3.0°
ω scans	*h* = −17→15
Absorption correction: multi-scan (*CrysAlis RED*; Oxford Diffraction, 2008)	*k* = −15→15
*T*_min_ = 0.955, *T*_max_ = 1.000	*l* = −15→14
17556 measured reflections	

### Refinement

**Table:**

Refinement on *F*^2^	Primary atom site location: structure-invariant direct methods
Least-squares matrix: full	Secondary atom site location: difference Fourier map
*R*[*F*^2^ > 2σ(*F*^2^)] = 0.045	Hydrogen site location: inferred from neighbouring sites
*wR*(*F*^2^) = 0.116	H-atom parameters constrained
*S* = 0.93	*w* = 1/[σ^2^(*F*_o_^2^) + (0.0687*P*)^2^] where *P* = (*F*_o_^2^ + 2*F*_c_^2^)/3
4169 reflections	(Δ/σ)_max_ = 0.002
319 parameters	Δρ_max_ = 0.34 e Å^−^^3^
0 restraints	Δρ_min_ = −0.25 e Å^−^^3^

### Special details

**Table:**

Geometry. All esds (except the esd in the dihedral angle between two l.s. planes) are estimated using the full covariance matrix. The cell esds are taken into account individually in the estimation of esds in distances, angles and torsion angles; correlations between esds in cell parameters are only used when they are defined by crystal symmetry. An approximate (isotropic) treatment of cell esds is used for estimating esds involving l.s. planes.

### Fractional atomic coordinates and isotropic or equivalent isotropic displacement parameters (Å^2^)

**Table:**

	*x*	*y*	*z*	*U*_iso_*/*U*_eq_	
C1	0.79663 (19)	0.93471 (19)	0.23882 (19)	0.0538 (6)	
H1	0.7585	0.9653	0.2766	0.065*	
C2	0.83868 (19)	0.9943 (2)	0.1808 (2)	0.0597 (7)	
H2	0.8295	1.0653	0.1783	0.072*	
C3	0.89659 (19)	0.9483 (2)	0.12396 (19)	0.0611 (7)	
H3	0.9250	0.9899	0.0836	0.073*	
C4	0.91234 (18)	0.8453 (2)	0.12618 (18)	0.0543 (7)	
H4	0.9516	0.8173	0.0883	0.065*	
C5	0.85827 (19)	0.50582 (19)	0.25407 (18)	0.0529 (6)	
H5	0.8967	0.4761	0.2162	0.064*	
C6	0.8169 (2)	0.4459 (2)	0.3131 (2)	0.0630 (7)	
H6	0.8270	0.3750	0.3146	0.076*	
C7	0.7591 (2)	0.4880 (2)	0.3722 (2)	0.0615 (7)	
H7	0.7319	0.4452	0.4127	0.074*	
C8	0.74323 (19)	0.58985 (19)	0.37013 (18)	0.0511 (6)	
H8	0.7052	0.6173	0.4098	0.061*	
C9	0.76717 (16)	0.76264 (17)	0.30277 (17)	0.0416 (6)	
N10	0.88297 (13)	0.67612 (15)	0.18987 (14)	0.0445 (5)	
C11	0.80948 (17)	0.82595 (17)	0.24349 (17)	0.0429 (6)	
C12	0.86933 (16)	0.78034 (18)	0.18595 (17)	0.0429 (6)	
C13	0.78353 (16)	0.65678 (17)	0.30837 (16)	0.0415 (6)	
C14	0.84322 (17)	0.61298 (17)	0.24995 (17)	0.0433 (6)	
C15	0.70287 (18)	0.80997 (17)	0.3623 (2)	0.0450 (6)	
O16	0.61194 (12)	0.81571 (14)	0.29809 (12)	0.0569 (5)	
O17	0.72938 (13)	0.83794 (15)	0.45268 (14)	0.0667 (5)	
C18	0.54272 (18)	0.8721 (2)	0.33483 (17)	0.0516 (6)	
C19	0.48032 (18)	0.8207 (2)	0.37892 (18)	0.0529 (7)	
C20	0.4108 (2)	0.8824 (2)	0.4042 (2)	0.0648 (8)	
H20	0.3666	0.8514	0.4338	0.078*	
C21	0.4053 (2)	0.9861 (3)	0.3872 (2)	0.0688 (8)	
H21	0.3582	1.0245	0.4057	0.083*	
C22	0.4685 (2)	1.0341 (2)	0.3430 (2)	0.0718 (8)	
H22	0.4648	1.1049	0.3313	0.086*	
C23	0.5384 (2)	0.9758 (2)	0.3157 (2)	0.0652 (8)	
H23	0.5817	1.0070	0.2849	0.078*	
C24	0.4878 (2)	0.7061 (2)	0.4025 (2)	0.0665 (8)	
H24	0.5264	0.6755	0.3599	0.080*	
C25	0.5415 (3)	0.6867 (3)	0.5193 (3)	0.1123 (14)	
H25A	0.6038	0.7197	0.5370	0.168*	
H25B	0.5500	0.6138	0.5315	0.168*	
H25C	0.5047	0.7143	0.5632	0.168*	
C26	0.3890 (3)	0.6532 (3)	0.3699 (3)	0.1142 (14)	
H26A	0.3566	0.6672	0.2959	0.171*	
H26B	0.3506	0.6789	0.4130	0.171*	
H26C	0.3976	0.5801	0.3803	0.171*	
C27	0.9445 (2)	0.6310 (2)	0.1287 (2)	0.0645 (7)	
H27A	0.9342	0.6677	0.0625	0.097*	
H27B	0.9277	0.5597	0.1139	0.097*	
H27C	1.0114	0.6362	0.1698	0.097*	
S28	0.91241 (5)	0.22174 (5)	0.57658 (5)	0.0556 (2)	
O29	0.94541 (16)	0.30956 (14)	0.53324 (15)	0.0809 (6)	
O30	0.87168 (17)	0.23980 (15)	0.66093 (15)	0.0838 (7)	
O31	0.97784 (15)	0.13502 (16)	0.59437 (16)	0.0819 (6)	
C32	0.8122 (2)	0.1755 (2)	0.4689 (2)	0.0603 (7)	
F33	0.73755 (14)	0.24221 (15)	0.44537 (15)	0.0955 (6)	
F34	0.77668 (13)	0.08743 (12)	0.49175 (13)	0.0840 (5)	
F35	0.83430 (13)	0.16316 (15)	0.37865 (11)	0.0895 (6)	

### Atomic displacement parameters (Å^2^)

**Table:**

	*U*^11^	*U*^22^	*U*^33^	*U*^12^	*U*^13^	*U*^23^
C1	0.0500 (17)	0.0489 (16)	0.0643 (15)	0.0028 (13)	0.0195 (14)	−0.0015 (12)
C2	0.0544 (18)	0.0509 (16)	0.0716 (17)	−0.0002 (13)	0.0151 (15)	0.0075 (14)
C3	0.0533 (19)	0.069 (2)	0.0583 (16)	−0.0096 (14)	0.0130 (14)	0.0150 (14)
C4	0.0429 (16)	0.0720 (19)	0.0499 (14)	−0.0010 (13)	0.0166 (12)	0.0044 (13)
C5	0.0505 (17)	0.0518 (16)	0.0532 (14)	0.0114 (13)	0.0101 (13)	−0.0025 (12)
C6	0.073 (2)	0.0457 (16)	0.0642 (16)	0.0109 (14)	0.0110 (15)	0.0028 (13)
C7	0.073 (2)	0.0538 (17)	0.0577 (15)	0.0001 (15)	0.0192 (15)	0.0078 (13)
C8	0.0516 (17)	0.0532 (16)	0.0500 (13)	0.0041 (12)	0.0175 (12)	0.0016 (12)
C9	0.0353 (15)	0.0456 (14)	0.0421 (12)	0.0012 (11)	0.0088 (11)	−0.0043 (10)
N10	0.0327 (12)	0.0512 (13)	0.0496 (11)	0.0048 (9)	0.0122 (10)	−0.0056 (9)
C11	0.0354 (14)	0.0459 (14)	0.0451 (12)	−0.0009 (11)	0.0079 (11)	−0.0033 (10)
C12	0.0313 (14)	0.0520 (16)	0.0419 (12)	0.0005 (11)	0.0057 (11)	−0.0020 (11)
C13	0.0350 (14)	0.0469 (14)	0.0400 (12)	0.0022 (11)	0.0072 (11)	−0.0027 (10)
C14	0.0352 (15)	0.0474 (15)	0.0433 (12)	0.0038 (11)	0.0053 (11)	−0.0011 (10)
C15	0.0448 (17)	0.0431 (14)	0.0491 (14)	0.0012 (11)	0.0168 (13)	−0.0002 (11)
O16	0.0371 (11)	0.0851 (13)	0.0496 (9)	0.0067 (9)	0.0144 (9)	−0.0141 (8)
O17	0.0565 (12)	0.0881 (14)	0.0508 (10)	0.0133 (10)	0.0083 (9)	−0.0208 (9)
C18	0.0415 (16)	0.0713 (18)	0.0416 (13)	0.0115 (13)	0.0117 (12)	−0.0051 (12)
C19	0.0424 (16)	0.0733 (18)	0.0438 (13)	0.0045 (13)	0.0141 (12)	−0.0088 (12)
C20	0.0462 (19)	0.088 (2)	0.0656 (16)	0.0073 (16)	0.0254 (14)	−0.0050 (15)
C21	0.053 (2)	0.092 (2)	0.0599 (17)	0.0258 (17)	0.0146 (15)	−0.0015 (16)
C22	0.073 (2)	0.075 (2)	0.0639 (17)	0.0230 (17)	0.0137 (16)	0.0102 (14)
C23	0.062 (2)	0.081 (2)	0.0571 (16)	0.0124 (16)	0.0234 (15)	0.0095 (14)
C24	0.062 (2)	0.0676 (19)	0.0780 (19)	−0.0062 (15)	0.0340 (16)	−0.0155 (15)
C25	0.169 (4)	0.072 (2)	0.092 (2)	−0.007 (2)	0.032 (3)	0.0148 (18)
C26	0.084 (3)	0.097 (3)	0.177 (4)	−0.025 (2)	0.062 (3)	−0.056 (3)
C27	0.0563 (19)	0.0703 (18)	0.0781 (17)	0.0122 (14)	0.0374 (15)	−0.0050 (14)
S28	0.0708 (5)	0.0511 (4)	0.0508 (4)	−0.0092 (4)	0.0269 (3)	−0.0037 (3)
O29	0.1012 (17)	0.0671 (12)	0.0837 (13)	−0.0311 (11)	0.0416 (13)	0.0000 (10)
O30	0.128 (2)	0.0756 (13)	0.0675 (11)	−0.0128 (12)	0.0596 (13)	−0.0138 (9)
O31	0.0703 (15)	0.0821 (14)	0.0873 (13)	0.0193 (12)	0.0142 (11)	0.0035 (11)
C32	0.061 (2)	0.0637 (18)	0.0655 (17)	0.0012 (15)	0.0326 (16)	0.0020 (13)
F33	0.0703 (13)	0.1043 (14)	0.1154 (14)	0.0249 (11)	0.0328 (11)	0.0228 (11)
F34	0.0793 (13)	0.0644 (11)	0.1129 (13)	−0.0209 (9)	0.0354 (11)	−0.0061 (9)
F35	0.0815 (13)	0.1338 (16)	0.0590 (9)	−0.0108 (11)	0.0300 (9)	−0.0252 (9)

### Geometric parameters (Å, °)

**Table:**

C1—C2	1.347 (3)	C18—C19	1.377 (3)
C1—C11	1.422 (3)	C19—C20	1.397 (3)
C1—H1	0.9300	C19—C24	1.515 (4)
C2—C3	1.405 (3)	C20—C21	1.362 (4)
C2—H2	0.9300	C20—H20	0.9300
C3—C4	1.354 (4)	C21—C22	1.365 (4)
C3—H3	0.9300	C21—H21	0.9300
C4—C12	1.412 (3)	C22—C23	1.389 (4)
C4—H4	0.9300	C22—H22	0.9300
C5—C6	1.353 (3)	C23—H23	0.9300
C5—C14	1.405 (3)	C24—C25	1.516 (4)
C5—H5	0.9300	C24—C26	1.527 (4)
C6—C7	1.405 (4)	C24—H24	0.9800
C6—H6	0.9300	C25—H25A	0.9600
C7—C8	1.340 (3)	C25—H25B	0.9600
C7—H7	0.9300	C25—H25C	0.9600
C8—C13	1.422 (3)	C26—H26A	0.9600
C8—H8	0.9300	C26—H26B	0.9600
C9—C11	1.389 (3)	C26—H26C	0.9600
C9—C13	1.391 (3)	C27—H27A	0.9600
C9—C15	1.507 (3)	C27—H27B	0.9600
N10—C12	1.365 (3)	C27—H27C	0.9600
N10—C14	1.373 (3)	S28—O30	1.4148 (17)
N10—C27	1.481 (3)	S28—O29	1.4161 (18)
C11—C12	1.431 (3)	S28—O31	1.443 (2)
C13—C14	1.428 (3)	S28—C32	1.799 (3)
C15—O17	1.188 (3)	C32—F35	1.321 (3)
C15—O16	1.335 (3)	C32—F34	1.322 (3)
O16—C18	1.431 (3)	C32—F33	1.344 (3)
C18—C23	1.366 (4)		
			
C2—C1—C11	121.2 (2)	C18—C19—C24	123.0 (2)
C2—C1—H1	119.4	C20—C19—C24	121.8 (2)
C11—C1—H1	119.4	C21—C20—C19	122.6 (3)
C1—C2—C3	119.5 (2)	C21—C20—H20	118.7
C1—C2—H2	120.2	C19—C20—H20	118.7
C3—C2—H2	120.2	C20—C21—C22	120.3 (3)
C4—C3—C2	122.0 (2)	C20—C21—H21	119.9
C4—C3—H3	119.0	C22—C21—H21	119.9
C2—C3—H3	119.0	C21—C22—C23	119.2 (3)
C3—C4—C12	120.1 (2)	C21—C22—H22	120.4
C3—C4—H4	119.9	C23—C22—H22	120.4
C12—C4—H4	119.9	C18—C23—C22	119.0 (3)
C6—C5—C14	120.1 (2)	C18—C23—H23	120.5
C6—C5—H5	120.0	C22—C23—H23	120.5
C14—C5—H5	120.0	C19—C24—C25	110.7 (2)
C5—C6—C7	121.7 (2)	C19—C24—C26	112.3 (3)
C5—C6—H6	119.2	C25—C24—C26	111.4 (3)
C7—C6—H6	119.2	C19—C24—H24	107.4
C8—C7—C6	119.9 (2)	C25—C24—H24	107.4
C8—C7—H7	120.1	C26—C24—H24	107.4
C6—C7—H7	120.1	C24—C25—H25A	109.5
C7—C8—C13	121.1 (2)	C24—C25—H25B	109.5
C7—C8—H8	119.4	H25A—C25—H25B	109.5
C13—C8—H8	119.4	C24—C25—H25C	109.5
C11—C9—C13	121.1 (2)	H25A—C25—H25C	109.5
C11—C9—C15	119.2 (2)	H25B—C25—H25C	109.5
C13—C9—C15	119.70 (19)	C24—C26—H26A	109.5
C12—N10—C14	122.16 (18)	C24—C26—H26B	109.5
C12—N10—C27	118.31 (19)	H26A—C26—H26B	109.5
C14—N10—C27	119.5 (2)	C24—C26—H26C	109.5
C9—C11—C1	122.5 (2)	H26A—C26—H26C	109.5
C9—C11—C12	118.9 (2)	H26B—C26—H26C	109.5
C1—C11—C12	118.6 (2)	N10—C27—H27A	109.5
N10—C12—C4	122.0 (2)	N10—C27—H27B	109.5
N10—C12—C11	119.5 (2)	H27A—C27—H27B	109.5
C4—C12—C11	118.5 (2)	N10—C27—H27C	109.5
C9—C13—C8	122.7 (2)	H27A—C27—H27C	109.5
C9—C13—C14	119.0 (2)	H27B—C27—H27C	109.5
C8—C13—C14	118.3 (2)	O30—S28—O29	116.47 (12)
N10—C14—C5	121.7 (2)	O30—S28—O31	113.97 (13)
N10—C14—C13	119.3 (2)	O29—S28—O31	114.20 (13)
C5—C14—C13	118.9 (2)	O30—S28—C32	104.08 (13)
O17—C15—O16	125.3 (2)	O29—S28—C32	104.00 (12)
O17—C15—C9	124.9 (2)	O31—S28—C32	101.75 (13)
O16—C15—C9	109.79 (19)	F35—C32—F34	108.1 (2)
C15—O16—C18	118.12 (17)	F35—C32—F33	105.2 (2)
C23—C18—C19	123.6 (2)	F34—C32—F33	105.7 (2)
C23—C18—O16	116.1 (2)	F35—C32—S28	112.96 (19)
C19—C18—O16	120.1 (2)	F34—C32—S28	112.72 (19)
C18—C19—C20	115.2 (2)	F33—C32—S28	111.7 (2)
			
C11—C1—C2—C3	−0.2 (4)	C8—C13—C14—N10	−179.9 (2)
C1—C2—C3—C4	−0.5 (4)	C9—C13—C14—C5	−178.8 (2)
C2—C3—C4—C12	0.6 (4)	C8—C13—C14—C5	1.2 (3)
C14—C5—C6—C7	−0.6 (4)	C11—C9—C15—O17	−92.5 (3)
C5—C6—C7—C8	0.5 (4)	C13—C9—C15—O17	87.2 (3)
C6—C7—C8—C13	0.4 (4)	C11—C9—C15—O16	87.4 (2)
C13—C9—C11—C1	−178.0 (2)	C13—C9—C15—O16	−92.8 (2)
C15—C9—C11—C1	1.7 (3)	O17—C15—O16—C18	8.9 (3)
C13—C9—C11—C12	1.2 (3)	C9—C15—O16—C18	−171.01 (19)
C15—C9—C11—C12	−179.1 (2)	C15—O16—C18—C23	86.1 (3)
C2—C1—C11—C9	179.9 (2)	C15—O16—C18—C19	−98.8 (3)
C2—C1—C11—C12	0.7 (4)	C23—C18—C19—C20	−0.3 (4)
C14—N10—C12—C4	178.4 (2)	O16—C18—C19—C20	−175.1 (2)
C27—N10—C12—C4	−0.4 (3)	C23—C18—C19—C24	−178.2 (2)
C14—N10—C12—C11	−1.9 (3)	O16—C18—C19—C24	7.0 (4)
C27—N10—C12—C11	179.4 (2)	C18—C19—C20—C21	−0.4 (4)
C3—C4—C12—N10	179.7 (2)	C24—C19—C20—C21	177.5 (3)
C3—C4—C12—C11	−0.1 (3)	C19—C20—C21—C22	0.6 (4)
C9—C11—C12—N10	0.5 (3)	C20—C21—C22—C23	0.0 (4)
C1—C11—C12—N10	179.7 (2)	C19—C18—C23—C22	0.8 (4)
C9—C11—C12—C4	−179.8 (2)	O16—C18—C23—C22	175.8 (2)
C1—C11—C12—C4	−0.6 (3)	C21—C22—C23—C18	−0.6 (4)
C11—C9—C13—C8	178.6 (2)	C18—C19—C24—C25	97.9 (3)
C15—C9—C13—C8	−1.2 (3)	C20—C19—C24—C25	−79.8 (3)
C11—C9—C13—C14	−1.4 (3)	C18—C19—C24—C26	−137.0 (3)
C15—C9—C13—C14	178.9 (2)	C20—C19—C24—C26	45.3 (3)
C7—C8—C13—C9	178.8 (2)	O30—S28—C32—F35	174.13 (19)
C7—C8—C13—C14	−1.2 (3)	O29—S28—C32—F35	51.7 (2)
C12—N10—C14—C5	−179.5 (2)	O31—S28—C32—F35	−67.2 (2)
C27—N10—C14—C5	−0.8 (3)	O30—S28—C32—F34	−63.0 (2)
C12—N10—C14—C13	1.6 (3)	O29—S28—C32—F34	174.54 (18)
C27—N10—C14—C13	−179.6 (2)	O31—S28—C32—F34	55.6 (2)
C6—C5—C14—N10	−179.2 (2)	O30—S28—C32—F33	55.7 (2)
C6—C5—C14—C13	−0.3 (3)	O29—S28—C32—F33	−66.7 (2)
C9—C13—C14—N10	0.0 (3)	O31—S28—C32—F33	174.42 (17)

### Hydrogen-bond geometry (Å, °)

**Table:**

*D*—H···*A*	*D*—H	H···*A*	*D*···*A*	*D*—H···*A*
C4—H4···O29^i^	0.93	2.48	3.363 (3)	159
C27—H27C···F35^i^	0.96	2.51	3.250 (4)	134

Symmetry codes:  (i) −*x*+2, *y*+1/2, −*z*+1/2.

### Table 2 S–O···π interactions (Å,°).

*Cg*1 and *Cg*2 are the centroids of the C9/N10/C11–C14 and C1–C4/C11/C12 rings, respectively.

**Table:**

*X*	*I*	*J*	*I*···*J*	*X*···*J*	*X*–*I*···*J*
S28	O29	*Cg*1^ii^	3.703 (2)	3.879 (2)	86.3 (1)
S28	O31	*Cg*1^ii^	3.528 (2)	3.879 (2)	92.9 (1)
S28	O31	*Cg*2^ii^	3.208 (2)	4.128 (2)	120.3 (2)

Symmetry code: (ii) –*x* + 2, –*y* + 1, –*z* + 1.

### Table 3 π–π interactions (Å,°).

**Table:**

*I*	*J*	*CgI*···*CgJ*	Dihedral angle	*CgI*_Perp	Cg*I*_Offset
3	3^iii^	3.962 (2)	0	3.340 (1)	2.131 (1)

Symmetry code: (iii) –*x* + 1, –*y* + 2, –*z* + 1.Notes: *Cg*3 is the centroid of the C18–C23 ring. Cg*I*···Cg*J* is the distance between ring centroids. The dihedral angle is that between the planes of the rings *I* and *J*. *CgI*_Perp is the perpendicular distance of *CgI* from ring *J*. Cg*I*_Offset is the distance between *CgI* and perpendicular projection of *CgJ* on ring *I*.
